# A glycosylation risk score comprehensively assists the treatment of bladder neoplasm in the real-world cohort, including the tumor microenvironment, molecular and clinical prognosis

**DOI:** 10.3389/fphar.2023.1280428

**Published:** 2023-09-25

**Authors:** Jinhui Liu, Yunbo He, Weimin Zhou, Zhuoming Tang, Zicheng Xiao

**Affiliations:** ^1^ Department of Urology, Xiangya Hospital, Central South University, Changsha, China; ^2^ National Clinical Research Center for Geriatric Disorders, Xiangya Hospital, Central South University, Changsha, China

**Keywords:** glycosylation, multi-omics, tumor heterogeneity, immunotherapeutic efficacy, molecular subtype, bladder carcinoma

## Abstract

**Background:** Bladder cancer is a common urological cancer associated high significant morbidity and mortality rates. Immunotherapy has emerged as a promising treatment option, although response rates vary among patients. Glycosylation has been implicated in tumorigenesis and immune regulation. However, our current comprehensive understanding of the role of glycosylation in bladder cancer and its clinical implications is limited.

**Methods:** We constructed a training cohort based on the downloaded TCGA-BLCA dataset, while additional datasets (Xiangya cohort, GSE32894, GSE48075, GSE31684, GSE69795 and E-MTAB-1803) from Xiangya hospital, GEO and ArrayExpress database were obtained and used as validation cohorts. To identify glycosylation-related genes associated with prognosis, univariate Cox regression and LASSO regression were performed. A Cox proportional hazards regression model was then constructed to develop a risk score model. The performance of the risk score was assessed in the training cohort using Kaplan-Meier survival curves and ROC curves, and further validated in multiple validation cohorts.

**Results:** We classified patients in the training cohort into two groups based on glycosylation-related gene expression patterns: Cluster 1 and Cluster 2. Prognostic analysis revealed that Cluster 2 had poorer survival outcomes. Cluster 2 also showed higher levels of immune cell presence in the tumor microenvironment and increased activation in key steps of the cancer immune response cycle. We developed an independent prognostic risk score (*p* < 0.001) and used it to construct an accurate prognostic prediction nomogram. The high glycosylation risk score group exhibited higher tumor immune cell infiltration, enrichment scores in immune therapy-related pathways, and a tendency towards a basal subtype. Conversely, the low-risk score group had minimal immune cell infiltration and tended to have a luminal subtype. These findings were consistent in our real-world Xiangya cohort.

**Conclusion:** This multi-omics glycosylation score based on these genes reliably confirmed the heterogeneity of bladder cancer tumors, predicted the efficacy of immunotherapy and molecular subtypes, optimizing individual treatment decisions.

## Introduction

Bladder cancer (BLCA) is a prevalent malignancy worldwide, characterized by high morbidity and mortality rates (Siegel et al., 2020). Non-muscle invasive bladder cancer (NMIBC) accounts for three-quarters of initial diagnoses, while the remaining cases are categorized as either muscle-invasive bladder cancer (MIBC) or BLCA with distant metastasis (Powles et al., 2022). Despite advancements in treatment options, such as surgery and chemotherapy, the prognosis for patients with advanced stage BLCA remains suboptimal (Antoni et al., 2017; Lenis et al., 2020). Recent studies have shown that monoclonal antibodies targeting PD-1 and its ligands have emerged as a therapeutic strategy with encouraging clinical benefits for metastatic BLCA (Rosenberg et al., 2016; Plimack et al., 2017; Rijnders et al., 2017; Hu et al., 2022). However, only a subset of patients can achieve beneficial (Jenkins et al., 2018; Schoenfeld and Hellmann, 2020). Although increased expression of PD-L1 on tumor cells and/or immune cells is currently used as a diagnostic method for immune therapies targeting PD-1, it only partially correlates with the clinical benefits of these drugs (Ma et al., 2016). Therefore, there is an urgent requirement to discover novel biomarkers that can assist in treatment decision-making and improve patient outcomes.

The tumor immune microenvironment (TIME) is an intricate milieu consisting of immune cells and immune-related molecules (Binnewies et al., 2018), and its importance in immunotherapy has gained widespread recognition. Chen DS et al. categorized TIME into three subtypes: “immune inflamed,” “immune excluded,” and “immune desert,” based on their distinct characteristics and potential responsiveness to immunotherapy (Chen and Mellman, 2017). Similarly, Duan Q et al. classified tumors as either “hot” or “cold” tumors depending on the level of immune infiltration (Duan et al., 2020). Cold and immune desert tumors are characterized by limited immune cell infiltration, resulting in a poor response to immunotherapy (Galon and Bruni, 2019). Emerging therapeutic strategies are focused on enhancing immune infiltration to transform the tumor microenvironment (TME), from a poorly infiltrated “cold” phenotype to an immune-rich “hot” phenotype (Bonaventura et al., 2019; Vonderheide, 2020). Therefore, analyzing the TIME is crucial for enhancing the efficacy of immunotherapy.

Glycosylation, a common post-translational protein modification process, occurs in all domains of life (Pinho and Reis, 2015). It involves the attachment of monosaccharides or polysaccharides (i.e., oligosaccharides or complex glycans) to specific residues of target proteins (Eichler, 2019). This modification has been reported to impact various biological processes, including protein secretion, degradation, transport to receptor interactions, and modulation of immune responses (Moremen et al., 2012; Varki, 2017). Glycosylation is associated with the pathogenesis of numerous prevalent diseases, including cancer (Eichler, 2019). Glycosylation modification affects tumorigenesis through its influence on growth, differentiation, metastasis, and immune surveillance. Altered glycosylation profiles have been detected in various types of cancer, including BLCA (Przybylo et al., 2002; RodrÍguez et al., 2018). For instance, the invasive capacity of BLCA cells has been linked to the N-glycosylation of cadherin (Przybylo et al., 2002). Furthermore, glycosylation has been implicated in the regulation of immune responses within the TIME (Badmann et al., 2020; Sun et al., 2021). However, our understanding of the glycosylation landscape in BLCA and its clinical implications is still limited. Given the critical role of glycosylation in both tumorigenesis and immune regulation, it is plausible that glycosylation patterns could serve as potential biomarkers for predicting the response to immunotherapy in BLCA patients.

In this study, our research objective is to develop a new glycosylation risk score based on a multi-omics study to evaluate the prognosis comprehensively and individually, immunophenotype, and tumor heterogeneity of BLCA patients. In addition, we aim to study the glycosylation risk score to provide valuable insights into the potential of BLCA patients to make treatment decisions such as personalized immunotherapy and improve their prognosis.

## Materials and methods

### Data collection

#### Training set

We established a dataset consisting of 408 patients with BLCA by selecting individuals from the Cancer Genome Atlas (TCGA) database. The mRNA expression matrix and clinical information corresponding to these patients were downloaded from the Genomic Data Commons (GDC, https://portal.gdc.cancer.gov/) (Colaprico et al., 2016). We converted the fragments per kilobase of exon model per million mapped fragments (FPKM) and count value in the original expression matrix to transcripts per kilobase of exon model per million mapped reads (TPM). Subsequently, we merged this data with clinical information to create a new dataset. After excluding 5 patients due to duplicated or missing follow-up data, a total of 403 patients formed the training cohort.

#### Validation cohorts

In our early-stage study (Li et al., 2021), we constructed a dataset called the Xiangya cohort and have uploaded it to the Gene Expression Omnibus (GEO) database. This dataset includes 56 patients with BLCA and encompasses complete survival information along with RNA-sequencing (RNA-seq) data (GSE188715). We also downloaded relevant data from the GEO database (https://www.ncbi.nlm.nih.gov/geo/) to construct four additional external validation cohorts (GSE32894, GSE48075, GSE31684 and GSE69795). Duplicate patients or those with incomplete survival information were excluded during data preprocessing, resulting in a final inclusion of 224 (GSE32894), 73 (GSE48075), 93 (GSE31684) and 38 (GSE69795) individuals in the four cohorts, respectively. Download the dataset with accession number E-MTAB-1803 from the ArrayExpress database (https://www.ebi.ac.uk/arrayexpress/) as an additional external validation cohort.

Supplementary Table S1 displays the clinical information of patients in the training and six validation cohorts.

### Consensus clustering

We obtained a list of 628 glycosylation-related genes from the gene set enrichment analysis (GSEA) (Supplementary Table S2). To analyze the expression pattern of these genes in training cohort, we utilized the consensus clustering function in the “ConsuClusterPlus” R package (Wilkerson and Hayes, 2010). The parameters were set as follows: distance = “manhattan”, clusterAlg = “pam”, maxK = 5, Reps = 1,500, pItem = 0.8, pFeature = 1. By applying this approach, we identified distinct glycosylation expression patterns.

### Describing the TIME of BLCA

To characterize the TIME of BLCA, we utilized the tracking tumor immunophenotype (TIP) database (http://biocc.hrbmu.edu.cn/TIP/) (Xu et al., 2018) to obtained the activation levels of the 7-step Cancer Immunity Cycle (CIC) (Chen and Mellman, 2013). Furthermore, we compiled a summary of 22 immune checkpoint inhibitor (ICI) genes, 18 T cell-associated inflammatory signature (TIS) genes, and effector genes of various immune cells, including CD8 T cells, dendritic cells (DCs), macrophages, natural killer (NK) cells, and type 1 T helper (Th1) cells, based on our previous study (Hu et al., 2021a) (Supplementary Table S3).

### Development of glycosylation risk score

To identify candidate genes associated with glycosylation patterns and clinical prognosis, we employed two methods: Univariate Cox analysis and the least absolute shrinkage and selection operator (LASSO) algorithm. The “glmnet” R package was utilized for the LASSO algorithm. Initially, Initially, we conducted univariate Cox analysis on a set of 628 genes and identified 30 genes that were strongly correlated with prognosis (*p* < 0.005). Subsequently, the LASSO algorithm was applied to further refine the prognostic genes. The “glmnet” R package facilitated this process. From the LASSO analysis, we identified 20 candidate genes. Finally, the glycosylation risk score was constructed using the Cox proportional hazard regression model with the “glmnet” R package, incorporating 20 genes.

$$Glycosylation\;Score=\sum \beta_{i}*{RNA}_{i}$$

### Evaluation and verification of glycosylation risk score

In the training set, patients were divided into high-risk and low-risk groups based on their risk scores, using the median of the risk score as the threshold. Kaplan-Meier (K-M) survival curves were plotted and the log-rank test was performed using the “survminer” R package to assess the differences in survival between the two groups. The predictive accuracy of the risk score was evaluated using the time-dependent receiver operating characteristic (tROC) analysis, implemented with the “tROC” R package. Additionally, a nomogram was constructed incorporating clinical information related to prognosis and the glycosylation risk score. The predictive efficacy of the nomogram was verified using calibration curves.

For external validation, the same method used in the training set was applied to an independent cohort of BLCA patients. In brief, the risk scores were calculated using the glycosylation risk scoring formula, and patients were classified into high-risk and low-risk groups using the median risk score as the threshold. The survival outcomes between the two groups were compared using the K-M method and log-rank test. The predictive accuracy of the risk scores was evaluated using tROC analysis.

### Identification of molecular subtypes of BLCA by glycosylation risk score

In our previous studies, our team conducted an extensive review and summary of the existing seven molecular typing criteria for BLCA, including the TCGA, UNC, and Consensus systems, et al. To achieve a unified classification approach, we utilized two R packages, namely, “BLCAsubtyping” and “ConsensusMIBC”. Additionally, we incorporated BLCA-related pathways identified by Kamoun, A. et al. (Kamoun et al., 2020) (Supplementary Table S4). To enhance clinical applicability, we further reclassified the different molecular subtypes into “luminal” and “basal” subtypes, aiming to provide a more concise and efficient clinical guidance.

### Statistical analysis

Correlations between variables were assessed using either Pearson or Spearman coefficients, depending on the nature of the data. Differences between binary groups in continuous variables were evaluated using the *t*-test or Mann-Whitney *U* test. To examine the survival prognosis, the K-M method was employed to generate survival curves, and statistical significance was determined using the log-rank test. The relationship between candidate genes and survival prognosis was determined through univariate Cox analysis, and the LASSO algorithm was used to select and refine the candidate genes for constructing the glycosylation risk score. The hazard ratio (HR) and independent prognostic values of the glycosylation risk score were calculated using univariate and multivariate Cox regression models. The glycosylation risk score was constructed using the Cox proportional hazard regression model, and its accuracy was assessed by drawing time-dependent receiver operating characteristic (ROC) curves and calculating the area under the curve (AUC). All statistical analyses were conducted using R software (version 4.22) with a significance level set at *p* < 0.05. The adjusted *p*-value was obtained using the false discovery rate (FDR) method, and all tests were two-sided.

## Results

### Construction of glycosylation genes expression patterns related to prognosis and tumor immune microenvironment

**FIGURE 1:**
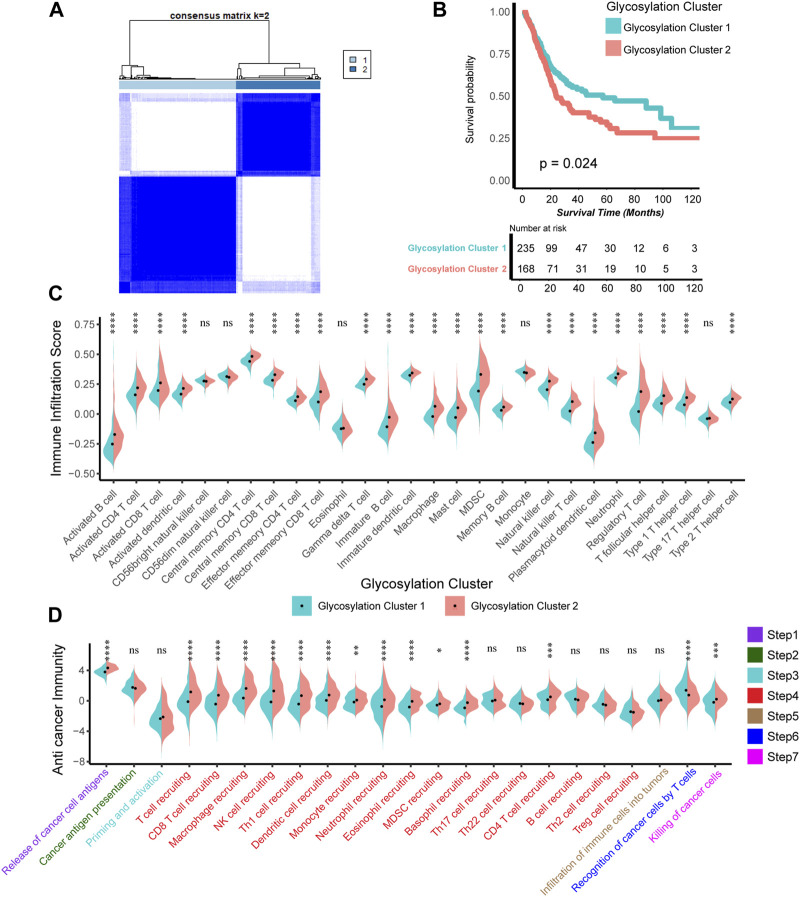
Construction of Glycosylation genes expression patterns related to prognosis and tumor immune microenvironment. **(A)** The unsupervised cluster analysis based on all the 628 Glycosylation-related genes; Light blue and dark blue lines represented Glycosylation cluster 1 and 2, separately. **(B)** Kaplan-Meier plot of OS between two Glycosylation-related patterns; Light green and red lines represented Glycosylation cluster 1 and 2, separately. **(C)** The different infiltration levels of 28 immune cells in the TME using ssGSEA algorithm between two Glycosylation-based patterns; Light green and red lines represent Glycosylation clusters 1 and 2, respectively. *p < 0.05, **p < 0.01, ***p < 0.001, ****p < 0.0001; ns, not statistically significant. **(D)** The different levels of anticancer immunity between two Glycosylation-based patterns; Light green and red lines represent Glycosylation cluster 1 and 2, respectively; *p < 0.05, **p < 0.01, ***p < 0.001, ****p < 0.0001; ns, not statistically significant.

We constructed expression patterns based on glycosylation genes features using unsupervised clustering analysis in the TCGA-BLCA cohort, by “ConsenseClusterPlus” R package. And we found that dividing into two patterns was the most appropriate, named glycosylation cluster 1 and glycosylation cluster 2. (Figure 1A). Subsequently, we conducted a detailed analysis to investigate the disparities between the two mentioned glycosylation clusters. In terms of prognosis, compared to cluster 1, cluster 2 has a significantly poorer prognosis (*p* = 0.024, Figure 1B). As for the TIME, as depicted in Figure 1C (Supplementary Table S5), the infiltration level of most of immune cells including activated and immature B cell, activated and central memory CD4 T cell, activated and central memory CD8 T cell, natural killer cell and macrophage in TME was apparently higher in cluster 2 compared to cluster 1. In addition, in the 7-step CIC, cluster 2 exhibited a higher activation level in the main anti-tumor immune steps, including step 1 (release of cancer cell antigens), step 4 (recruitment of immune cells such as T cell, CD8 T, macrophage, NK cell, dendritic cell), step 6 (recognition of cancer cells by T cells) and step 7 (killing of cancer cells) (Figure 1D; Supplementary Table S6).

### Developing glycosylation-related risk scores and predicting clinical outcomes in multiple cohorts

The completely different manifestations of these two glycosylation clusters mentioned above in the prognosis and TIME of BLCA aroused our interest. Therefore, we planned to develop a quantitative risk score utilizing the expression patterns of glycosylation genes. This risk score will be used to predict the clinical prognosis of each patient, aiming to achieve the accuracy treatment of BLCA.

**FIGURE 2:**
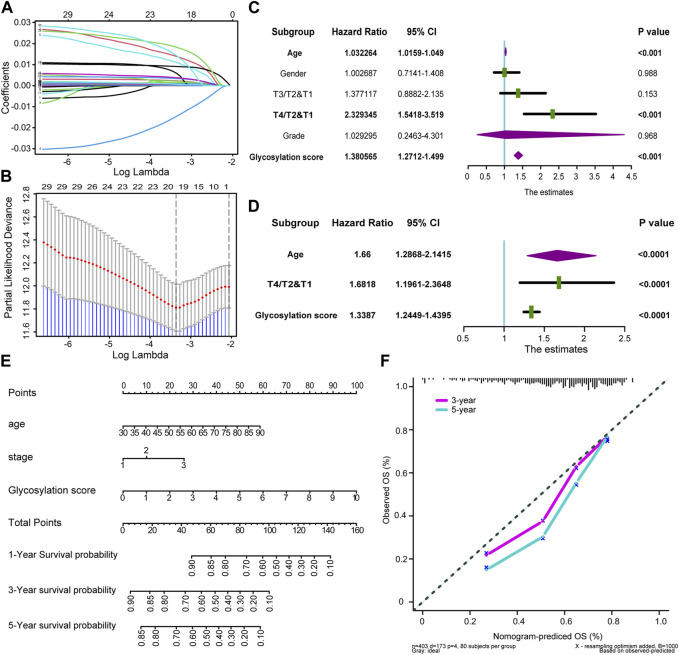
Developing Glycosylation-related risk scores and predicting clinical outcomes in multiple cohorts **(A)** Coefficients of Glycosylation-related prognosis genes value are shown by lambda parameter. **(B)** Partial likelihood deviance versus log (lambda) drawn by LASSO algorithm and 10-fold cross-validation. **(C,D)** Forest plots of univariate and multivariate Cox analysis of Glycosylation-based risk score combined with age, gender, tumor grade and stage of BLCA. **(E)** Nomogram developed by using age, tumor stage, and Glycosylation-based risk score. **(F)** Calibration curves of the nomogram.

Firstly, we selected 30 independent prognostic genes strongly associated with prognosis from glycosylation-related genes through univariate analysis (*p* < 0.005, Supplementary Table S7). Subsequently, LASSO regression helped us identify the 20 most suitable candidate genes in those 30 independent prognostic genes above for constructing glycosylation-related risk models (Figures 2A, B; Supplementary Table S8). And we choose the minimum lambda for the optimal cutoff value, and selected ten-fold cross validation method as correction. Finally, based on those 20 candidate glycosylation genes above, we employed the “glmnet” R software package to construct a Cox proportional risk regression model. This model allowed us to generate a risk score, known as the glycosylation-based risk score, in the TCGA-BLCA training cohort. The median glycosylation risk score would be used as a standard to classify the patients in cohorts into high and low score groups.

To investigate the potential clinical utility of glycosylation risk score, we first included it as an independent clinical indicator through univariate COX analysis. Our findings revealed that the glycosylation risk score, along with other clinical pathological factors such as tumor grade, stage of BLCA, age, and gender, significantly influenced prognosis (*p* < 0.001, Figure 2C). As shown in Figure 2D, subsequent multivariate Cox analysis demonstrated that the glycosylation risk score remains an independent prognostic indicator (*p* < 0.001). A glycosylation specific nomogram was developed using those independent prognostic factors identified by multivariate COX analysis (glycosylation risk score, age, and tumor stage) suggested that glycosylation risk score, like other clinical information, have important predictive value for prognosis (Figure 2E). The results indicated that glycosylation score accounted for a considerable proportion of the nomogram score, so its level could largely accurately predict the survival probability of a single patient at 1, 3, and 5 years. Furthermore, it was found that patients with higher glycosylation score had poorer prognosis, and there was an urgent need for new treatment methods to improve prognosis of these patients. In addition, the calibration curve indicated that the predicted OS of the glycosylation specific nomogram was very close to the real OS (Figure 2F), and the Q-Q plot had verified the normality of the above data (Supplementary Figure S1).

**FIGURE 3:**
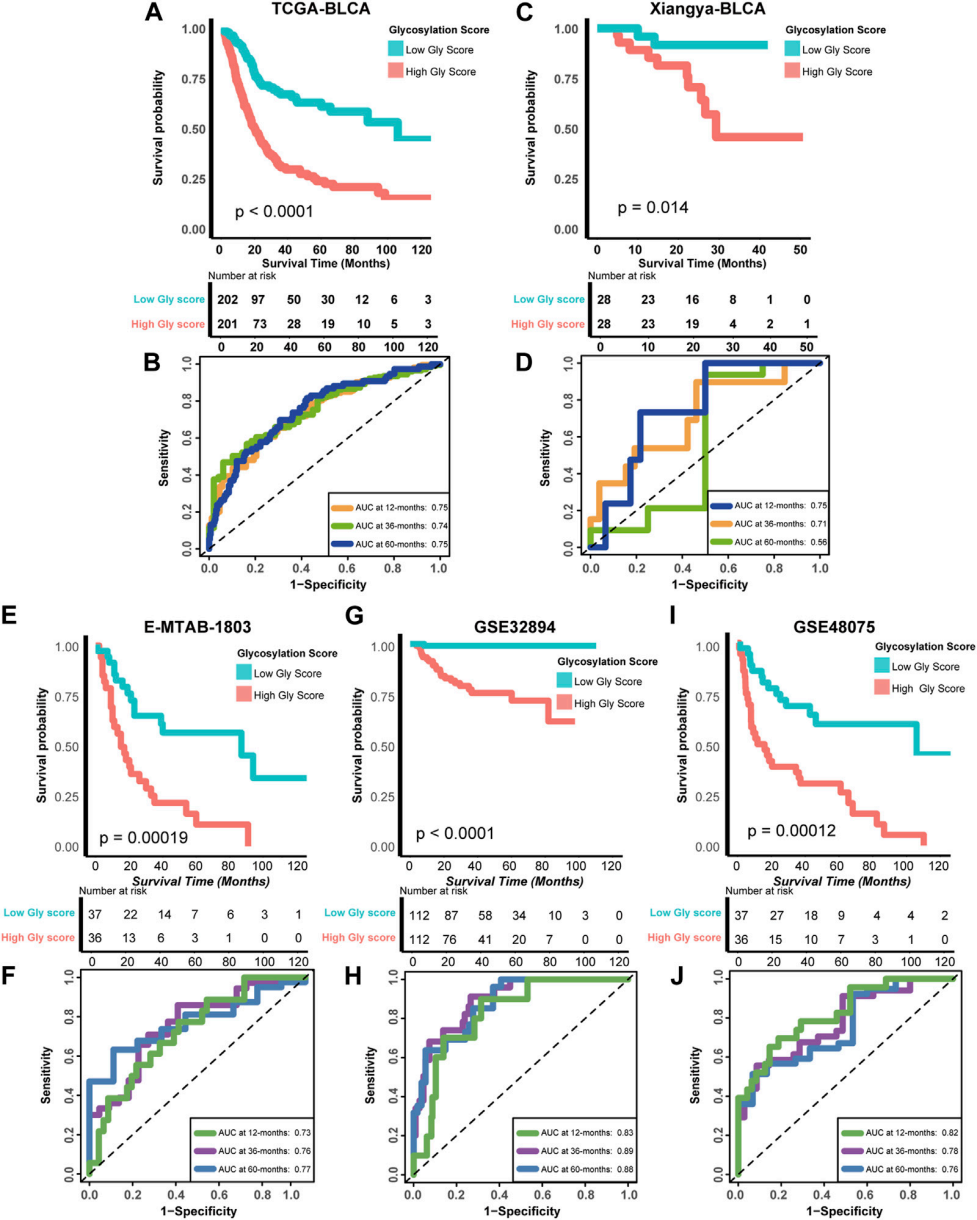
Verifying the accuracy of Glycosylation score in predicting prognosis in multiple cohorts **(A)** Kaplan-Meier (K–M) plot of OS between Glycosylation risk score groups in TCGA-BLCA cohort; Light red and green lines represented high and low Glycosylation risk score groups, separately. **(B)** The area under curves (AUCs) plot of Glycosylation risk score in TCGA-BLCA cohort. **(C,D)** K-M plot of OS between Glycosylation risk score groups and AUCs plot of the risk score the in Xiangya validation cohort, separately. **(E,F)** K-M plot of OS between Glycosylation risk score groups and AUCs plot of the risk score the in E-MTAB-1803 validation cohort, separately. **(G,H)** K-M plot of OS between Glycosylation risk score groups and AUCs plot of the risk score the in GSE32894 validation cohort, separately. **(I,J)** K-M plot of OS between Glycosylation risk score groups and AUCs plot of the risk score the in GSE48075 validation cohort, separately.

To further determine the prognostic significance of glycosylation risk score in BLCA, we validated its predictive value in multiple cohorts, including both public databases and our own real-world study. In the training cohort TCGA-BLCA, we observed that patients with a high glycosylation risk score had significantly worse prognosis compared to those with a low glycosylation risk score (*p* < 0.0001, Figure 3A). Additionally, the glycosylation risk score exhibited high accuracy in predicting 1-year, 3-year, and 5-year survival rates, with respective values of 0.75, 0.74, and 0.75 (Figure 3B). Meanwhile, in our real-world cohort (Xiangya BLCA cohort), the prognosis of the high glycosylation score group remained significantly poor (*p* = 0.014, Figure 3C) and its predictive accuracy was relatively high (1, 3, and 5 years accuracy: 0.75, 0.71 and 0.56 separately, Figure 3D). The above results remain robust: the prognosis of the high glycosylation score group presented obviously worse, in other public database cohorts, including E-MTAB-1803 (*p* = 0.00019, 1-year, 3-year, and 5-year accuracy: 0.73, 0.76 and 0.77 separately, Figures 3E, F), GSE32894 (*p* < 0.0001, 1-year, 3-year, and 5-year accuracy: 0.83, 0.89 and 0.88 separately, Figures 3G, H), GSE48075 (*p* = 0.00012, 1-year, 3-year, and 5-year accuracy: 0.82, 0.78 and 0.76 separately, Figures 3I, J), and two other GEO BLCA cohorts (Supplementary Figure S2).

The above results fully confirmed that glycosylation risk score can reliably predict the clinical outcomes of BLCA, and its predictive value had high accuracy and internal and external authenticity, which can be widely promoted to other cohorts.

### Exploring the relationship between glycosylation score and TIME in the TCGA-BLCA cohort

**FIGURE 4:**
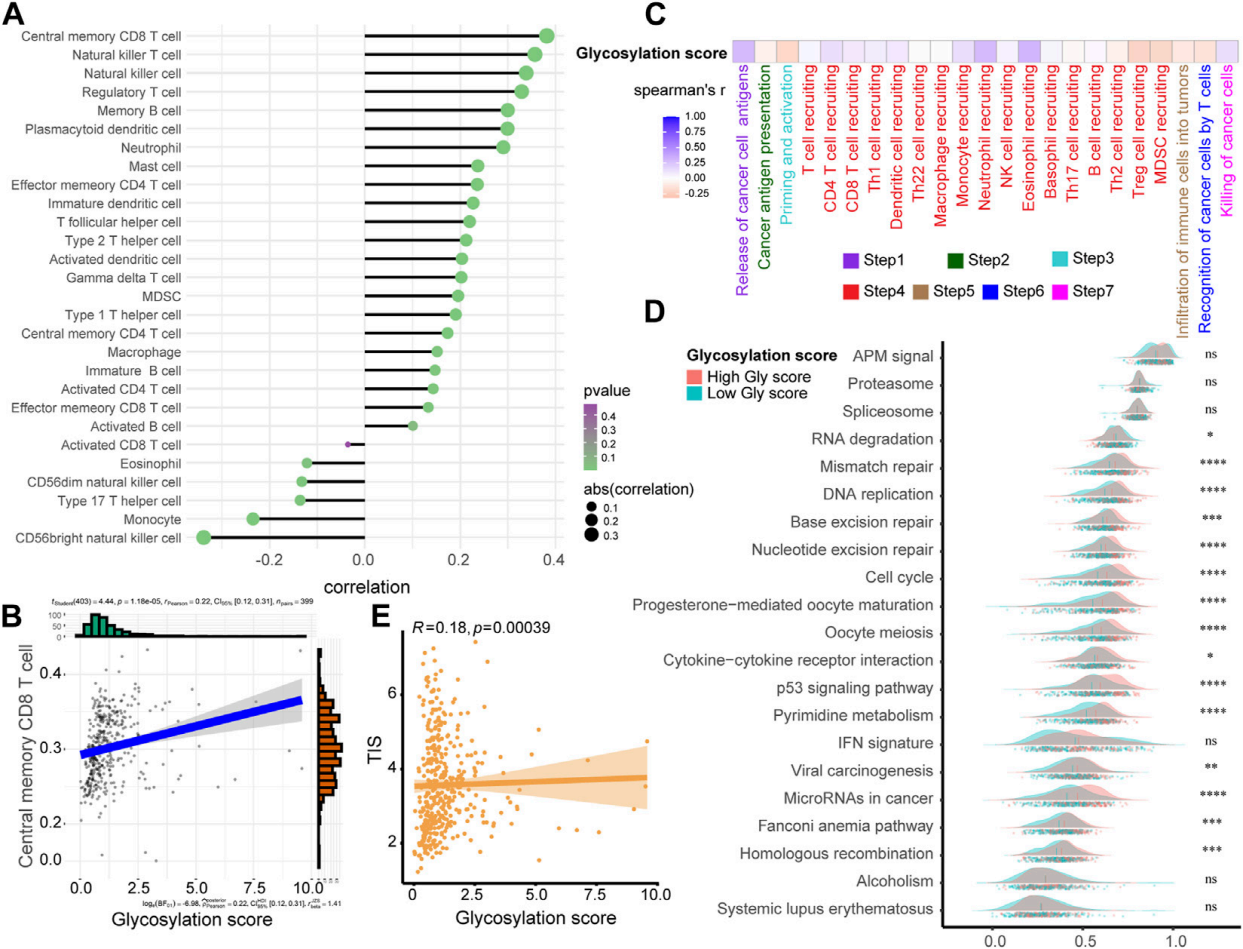
Exploring the relationship between Glycosylation score and TIME in the TCGA-BLCA cohort. **(A)** The association between Glycosylation risk score and immune cells in the Glycosylation in the TCGA-BLCA cohort, high Glycosylation score vs. low Glycosylation score. **(B)** The relationship between central memory CD8 T cells and Glycosylation score. **(C)** The association between Glycosylation risk score and cancer immunity cycles in the TCGA-BLCA cohort. **(D)** The different activated levels of gene signatures associated with ICB response between different Glycosylation-based risk score groups. Light red and green lines represent high and low Glycosylation-based risk score, respectively; *p < 0.05, **p < 0.01, ***p < 0.001, ****p < 0.0001; ns, not statistically significant. **(E)** The association between Glycosylation-based risk score and T cell-associated inflammatory signature (TIS) score.

The accurate prediction of glycosylation score for prognosis had sparked our interest in deeper research, therefore, we continued to investigate its association with the TIME in the TCGA-BLCA cohort. As shown in the single-sample gene set enrichment analysis (ssGSEA) analysis (Figure 4A; Supplementary Table S9), we found that compared to the low glycosylation score patients, the infiltration level of major tumor immune cells significantly increased in patients with high glycosylation score, such as central memory CD8/CD4 T cell, natural killer T cell, natural killer cell, regulatory T cell and memory B cell. The correlation between representative immune cells infiltration and glycosylation score were shown in Figure 4B and Supplementary Figure S3, Meanwhile, the activation of major steps of 7-step CIC, such as release of cancer cell antigens, immune cells recruiting and killing of cancer cells, was significantly higher in patients with high glycosylation score than those with low glycosylation score (Figure 4C; Supplementary Table S10). Furthermore, we examined the relationship between the glycosylation risk score and the enrichment score of gene features related to 21 immunotherapy-related pathways that summarized by Mariathasan et al. (2018). The findings revealed that the glycosylation score group exhibited higher pathway enrichment scores (Figure 4D). Finally, based on the TIS score of predicting immune checkpoint blocker (ICB) efficacy summarized by our team’s previous research, we observed that the patients with higher glycosylation score also had higher TIS scores (Figure 4E). The consistency of the above results indicated that the high glycosylation score group was more inclined to express the “hot-immune” TME, and was predicted to be more sensitive to immunotherapy.

### Verifying the relationship between glycosylation score and TIME in a real-world BLCA cohort

**FIGURE 5:**
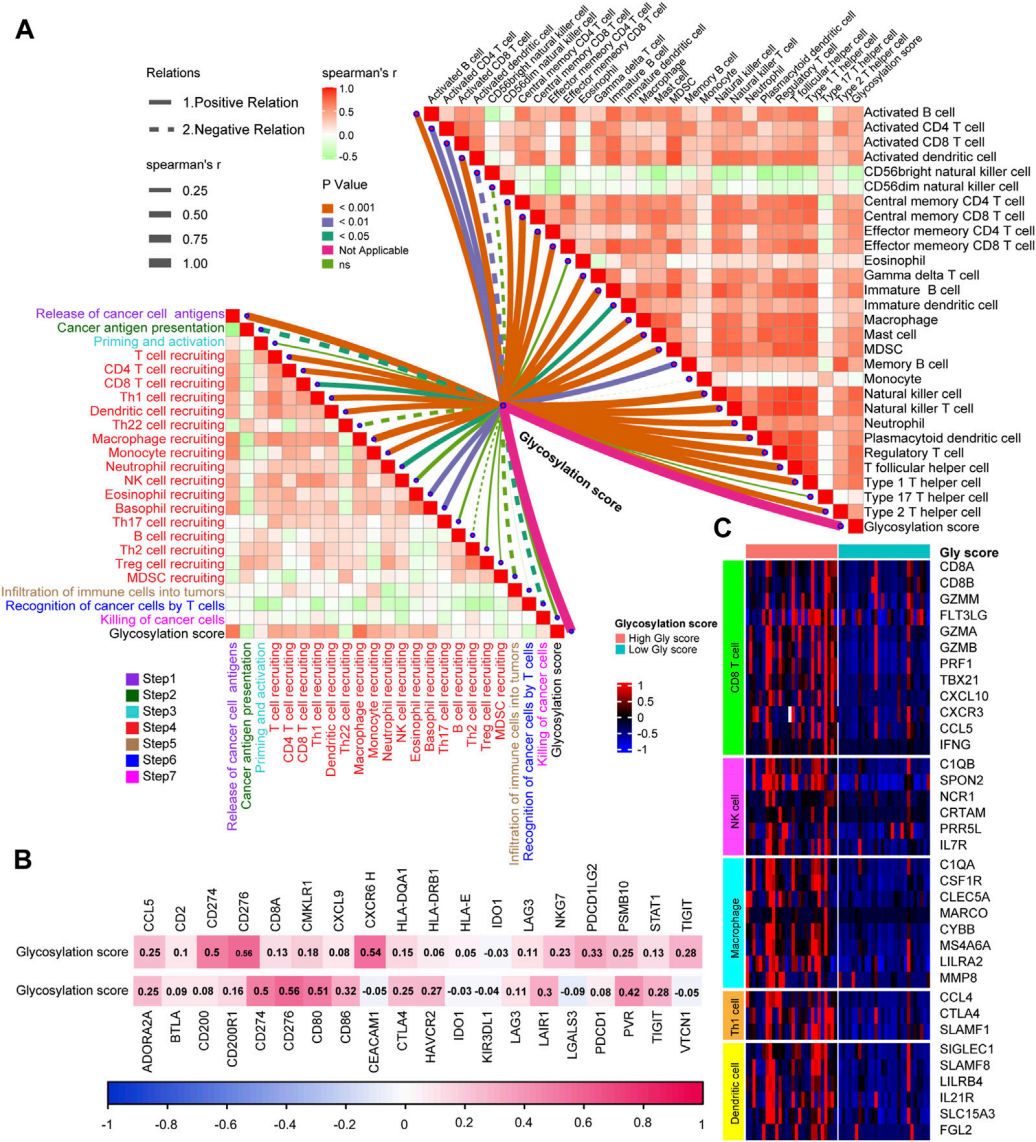
Verifying the relationship between Glycosylation score and TIME in a real-world BLCA cohort. **(A)** The association between Glycosylation-based risk score and cancer immunity cycles (left) and immune cells in the TME (right). The different types of lines represent the positive or negative relations. The different colors of the lines represent the *p* values of the relations, and the thickness of the lines represents the strength of the relations. **(B)** The association between Glycosylation risk score and T cell-associated inflammatory signature (TIS) genes (up) and immune checkpoint inhibitor (ICI) genes (down), separately. **(C)** The different expression patterns of effector genes of immune cells between different Glycosylation risk score groups, Light red and green lines represented high and low Glycosylation risk score groups, separately.

Based on glycosylation scores to predict the expression of TME immunophenotype in TCGA-BLCA, we verified how glycosylation affects the TIME in real BLCA patients in Xiangya Hospital. Like previous research ideas, in 7-step CIC (Figure 5A, left; Supplementary Table S11), we found that patients with high glycosylation scores were more activated in the main anti-tumor immune steps, including. Correspondingly, the level of immune cell infiltration in the TIME was significantly increased in patients with high scores (Figure 5A, right; Supplementary Table S12), including. Furthermore, as shown in Figure 5B (Supplementary Table S13), patients with high glycosylation scores also expressed more ICI (up) and TIS (down) related genes, further confirming the activity of immune cells in their TME. As for immune cell effector genes, the results strongly suggested that patients with higher glycosylation score express more effector genes for CD8+T cells, DC, macrophages, NK cells, and Th1 cells (Figure 5C). Based on the multiple verifications of glycosylation and immunity in TCGA-BLCA and Xiangya-BLCA, we conclude that patients with higher glycosylation scores often exhibited a “hot” TIME, which mad their efficacy in immunotherapy more ideal.

### Glycosylation score guided precision medicine in BLCA by predicting molecular subtypes

The gene expression profiling of MIBC has revealed that it was a heterogeneous disease, that can be sub-grouped into a variety of molecular subtypes, and shared significantly different prognoses and responses to anti-tumor treatments (Sjödahl et al., 2012; Comprehensive molecular characterization of urothelial, 2014; Fu et al., 2018; McConkey and Choi, 2018). The most common and recognized molecular typing standards were as follows, consensus subtype (Kamoun et al., 2020), TCGA subtype (Robertson et al., 2017), Cartes d’Identité des Tumeurs-Curie (CIT) subtype (Rebouissou et al., 2014), Lund subtype (Marzouka et al., 2018), Baylor subtype (Mo et al., 2018), University of North Carolina (UNC) subtype (Damrauer et al., 2014), MDAnderson Cancer Center (MDA) subtype (Choi et al., 2014). Our previous research integrated and simplified the above 7 typing standards to promote the clinical implementation of BLCA molecular subtypes (Li et al., 2021).

**FIGURE 6:**
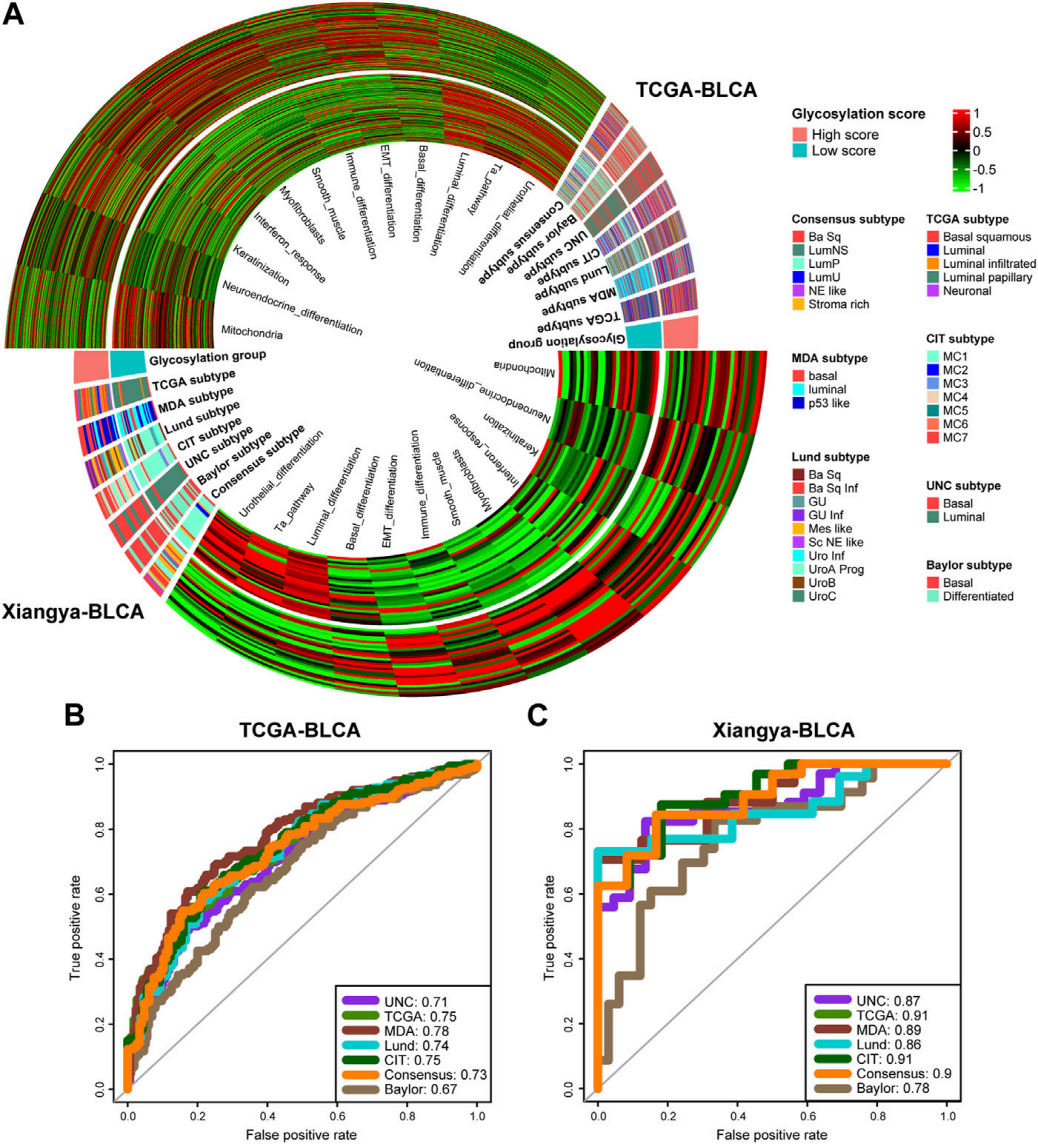
Glycosylation score guided precision medicine in BLCA by predicting molecular subtypes. **(A)** The heatmap of different Glycosylation risk score groups, seven molecular subtype classifications and bladder cancer associated signatures in the TCGA-BLCA (up) and Xiangya cohort (down). Activated or inhibited pathways are marked as red or green, separately. **(B,C)** ROC plot of the Glycosylation risk score for predicting seven molecular subtype classifications in BLCA in the TCGA-BLCA and Xiangya cohort.

We found high consistency in the results between the public training cohort TCGA-BLCA (Figure 6A, up) and our real-world research cohort Xiangya-BLCA (Figure 6A, down). Among all the classification criteria, the basal subtype was more inclined to obtain higher glycosylation score, while the luminal subtype was more inclined to obtain lower glycosylation score. And, patients with high glycosylation score tend to exhibit basal differentiation characteristics, such as EMT differentiation, Immune differentiation, basal differentiation, interferon response, and so on. Simultaneously, patients in the with low glycosylation score were more inclined to exhibit luminal differentiation, like luminal differentiation and urothelial differentiation. As for the accuracy of glycosylation score in predicting BLCA molecular subtype, in TCGA-BLCA (Figure 6B), most AUCs exceed 0.73, and in Xiangya BLCA (Figure 6C), most AUCs even exceed 0.87.

Previous studies (Seiler et al., 2017; Kamoun et al., 2020) have shown that the differentiation of the Basal subtype BLCA is lower than that of the Luminal subtype, resulting in poorer prognosis. However, it has a higher response rate to immunotherapy such as cisplatin and ICB. Our study showed that BLCA patients with high glycosylation score had poor prognosis but more immune cell infiltration due to a tendency towards lower differentiated Basal subtype.

## Discussion

Neoadjuvant chemotherapy based on cisplatin, followed by radical cystectomy and urinary tract diversion, remains the standardized treatment plan for locally advanced MIBC since the early 21st century (Author Anonymous, 1999; Grossman et al., 2003). Glycosylation is involved in many fundamental cellular events, including cell migration, cell signaling, growth and intercellular adhesion, cell signaling, and growth, and is one of the most common post translational modifications of proteins (Fuster and Esko, 2005). And, abnormal glycosylation is also considered an indispensable part of the carcinogenesis process (Ni et al., 2014), including BLCA. The research on protein modification and tumor heterogeneity, as well as the prediction of immunotherapy efficacy such as ICB, remains a hot topic in many cancers (Liu et al., 2021; Liu et al., 2022). Therefore, our research was dedicated to deeply exploring the association between glycosylation and BLCA, with the goal of accurately predicting prognosis and individualized guidance for treatment of BLCA.

Firstly, based on the expression feature of 628 glycosylation genes in each TCGA-BLCA patient, we obtained the most appropriate two clusters through consensus clustering, named glycosylation cluster 1 and glycosylation cluster 2. The results indicated that the patients in glycosylation cluster 2 had poor prognosis but more immune cell infiltration into the TME. The poor prognosis and unsatisfactory treatment response of cancer are mostly related to complex TME (Siegel et al., 2021), with the role of immune cells and related pathways being important factors. Therefore, we hoped to further develop the quantitative value of glycosylation genes in predicting prognosis and immunophenotype of BLCA. In addition, in recent years, research on the mechanism of glycosylation in BLCA had made progress (Wu et al., 2021; Tan et al., 2022), but research on the development of risk score to evaluate the prognosis of BLCA was still lacking. Therefore, we constructed a model by screening candidate genes that were strongly correlated with prognosis and most representative of glycosylation gene expression characteristics, and for the first time developed a glycosylation risk score that can comprehensively predict the prognosis, immune phenotype, and molecular subtype of BLCA.

Tumor cells had a faster rate of protein glycosylation than normal cells (Beatson et al., 2016), and a prospective multi-omics study on ovarian cancer by Hu et al. (2020). Further demonstrated that there was a significant differential expression of glycosylation between cancer cells and normal cells, and the degree of glycosylation difference could be reflected by the expression of glycoproteins in cancer cells. Some studies had shown that the abnormal mutation of the glycosylation related gene GALNT1 would lead to the occurrence and progression of a variety of cancers, including BLCA (Dyrskjøt et al., 2009). The activity of tumor infiltrating immune cells (TIICs), especially tumor infiltrating lymphocytes (TIL), in TME directly determines the survival outcome of tumor patients (Fridman et al., 2012), including early pT1 BLCA (Hülsen et al., 2020). In addition, the development of new targets, such as BCAT2, EMT-related signature and S100A5 (Xiao et al., 2022; Cai et al., 2023; Li et al., 2023), was playing an increasingly important role in immunotherapy for BLCA. A multicenter cohort study involving 709 patients (Bajorin et al., 2021) suggested that BLCA patients who still had a high risk of recurrence after surgery should be assisted with nivolumab. In this study, patients with high glycosylation scores had worse prognosis but presented a “hot” TIME (Duan et al., 2020) with high immune cell infiltration, and this result was highly consistent in the training set TCGA-BLCA and our own real-world cohort Xiangya-BLCA. Badmann S. et al.'s study (Badmann et al., 2020) provided a possible explanation for this phenomenon: in ovarian cancer, glycosylation could promote macrophage differentiation towards anti-inflammatory M2 type, leading to immune escape of cancer cells in immune activated TME.

Previous studies had shown that molecular typing can refine the prognosis and immune microenvironment of tumors. For example, in the study of breast cancer, it was found that the TILs infiltration level of different molecular subtypes of HR + breast cancer was quite different, in which TILs infiltration only prolongs OS, not disease-free survival (DFS) (Denkert et al.,2018). That was to say, different molecular subtypes exhibit different TIME (Goldberg et al., 2021). Rethinking the criteria for tumor molecular typing had become a hot topic, such as refining, updating, integrating, and simplifying. For example, the 5 mC regulator subtype system developed by our team in the previous study can accurately predict molecular typing in BLCA (Hu et al., 2021b). Moreover, the establishment of a consensus molecular subtype standard in gastric adenocarcinoma (GAC) to reclassify it and predict the response rate to ICB treatment (Wu et al., 2022), and a new standard developed by our team (Li et al., 2021) that integrate multiple mainstream molecular subtypes of BLCA will bring molecular typing closer to tumor treatment practice. In addition, Miao et al. (2022) reported that a glycosylation related protein B3GNT5 was specifically overexpressed in basal-like breast cancer (BLBR), revealing the close relationship between glycosylation and cancer molecular subtype. In this study, BLCA patients with high glycosylation score tended to differentiate into basal subtype, and they had “hot” TIME characteristics, but had poor prognosis. However, patients with low glycosylation score exhibited opposite luminal subtype, as well as corresponding prognosis and immune phenotype. In summary, patients with high glycosylation score would have better expected efficacy in receiving immunotherapy such as ICB, so more efforts should be made to explore new immunotherapies to improve the prognosis after treatment. On the contrary, patients with low glycation score should focus more on the development of targeted therapies and other therapies. This result also confirmed the previous research on the impact of molecular subtype on tumor prognosis and immunity phenotype (Choi et al., 2014; Hodgson et al., 2018).

Finally, there are some limitations that need to be further explored and supplemented in future research in this study. First, the materials of this study were retrospective data, and the influence between glycosylation and prognosis, immunophenotype and molecular typing of BLCA mostly stops at the level of correlation. Therefore, we plan to take this study as a pre-study and carry out prospective research on glycosylation and immunotherapy and targeted therapy of BLCA in the follow-up series of studies. In addition, based on this study and more literature review, combined with experimental conditions, we will conduct research on the mechanism of glycosylation related genes affecting BLCA treatment, committed to developing new therapeutic targets to promote precise treatment of BLCA.

## Conclusion

Our study constructed a glycosylation score related to BLCA through multi-omics data, and predicted the tumor heterogeneity, prognosis and immunophenotype of BLCA. Glycosylation score can reliably predict the efficacy of immunotherapy and molecular subtypes of BLCA, which is conducive to individualized treatment decisions of BLCA patients.

## Figures and Tables

**Table:** 

**BLCA**	bladder cancer
**NMIBC**	non-muscle invasive bladder cancer
**MIBC**	muscle-invasive bladder cancer
**TIME**	tumor immune microenvironment
**TME**	tumor microenvironment
**TCGA**	the Cancer Genome Atlas
**GDC**	Genomic Data Commons
**FPKM**	fragments per kilobase of exon model per million mapped fragments
**TPM**	transcripts per kilobase of exon model per million mapped reads
**GEO**	Gene Expression Omnibus
**RNA-seq**	RNA-sequencing
**GSEA**	gene set enrichment analysis
**TIP**	tracking tumor immunophenotype
**CIC**	Cancer Immunity Cycle
**ICI**	immune checkpoint inhibitor
**TIS**	T cell-associated inflammatory signature
**DCs**	dendritic cells
**NK**	natural killer
**Th1**	type 1 T helper
**LASSO**	least absolute shrinkage and selection operator
**K-M**	Kaplan-Meier
**tROC**	time-dependent receiver operating characteristic
**HR**	hazard ratio
**ROC**	receiver operating characteristic
**AUC**	area under the curve
**ssGSEA**	single sample gene set enrichment analysis
**ICB**	immune checkpoint blocker
**TIICs**	tumor infiltrating immune cells
**TIL**	tumor infiltrating lymphocytes
**DFS**	disease-free survival
**GAC**	gastric adenocarcinoma
**BLBR**	basal-like breast cancer

## Data Availability

The datasets presented in this study can be found in online repositories. The names of the repository/repositories and accession number(s) can be found in the article/Supplementary Material.
