# Supplementary figures and images for: A glycosylation risk score comprehensively assists the treatment of bladder neoplasm in the real-world cohort, including the tumor microenvironment, molecular and clinical prognosis
Source: Front Pharmacol. 2023 Sep 25;14:1280428. doi: 10.3389/fphar.2023.1280428 (PMC10560734; doi:10.3389/fphar.2023.1280428)

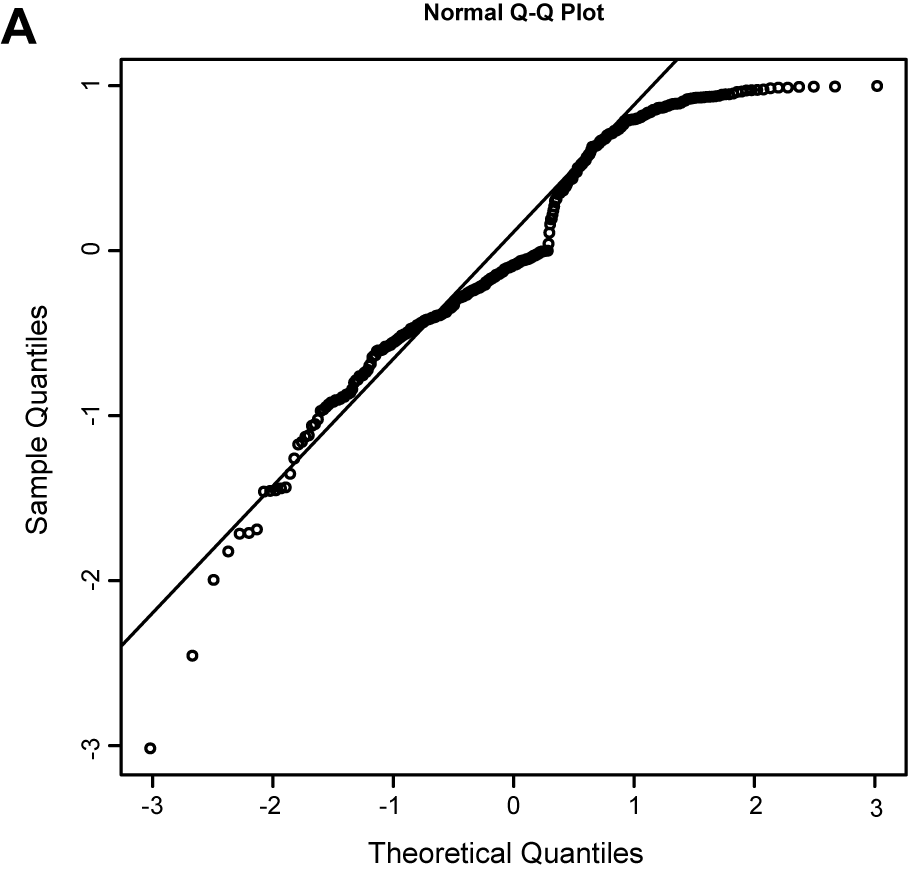

Supplement: Supplementary file 4 [file Image3.TIF]

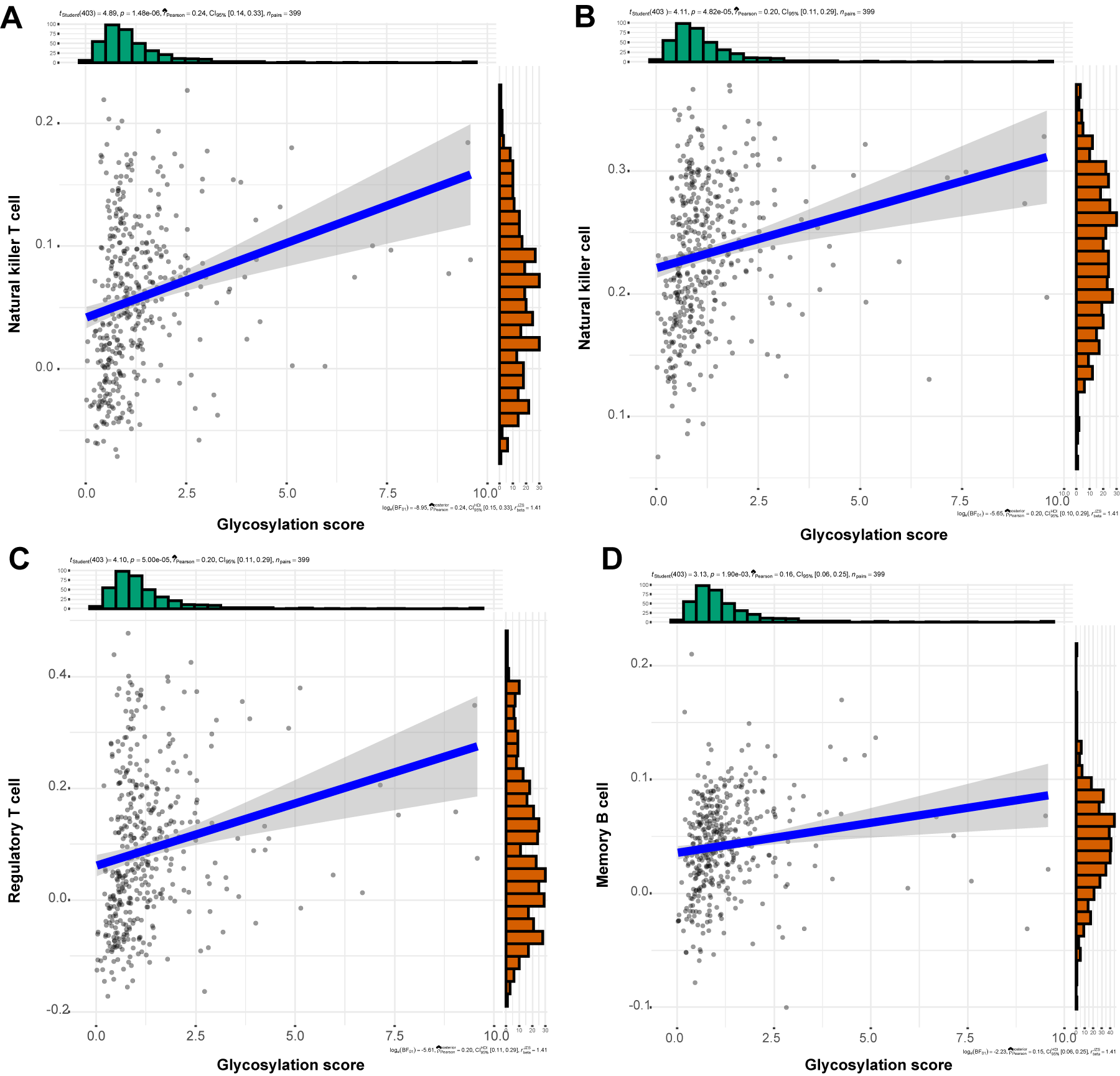

Supplement: Supplementary file 5 [file Image2.TIF]

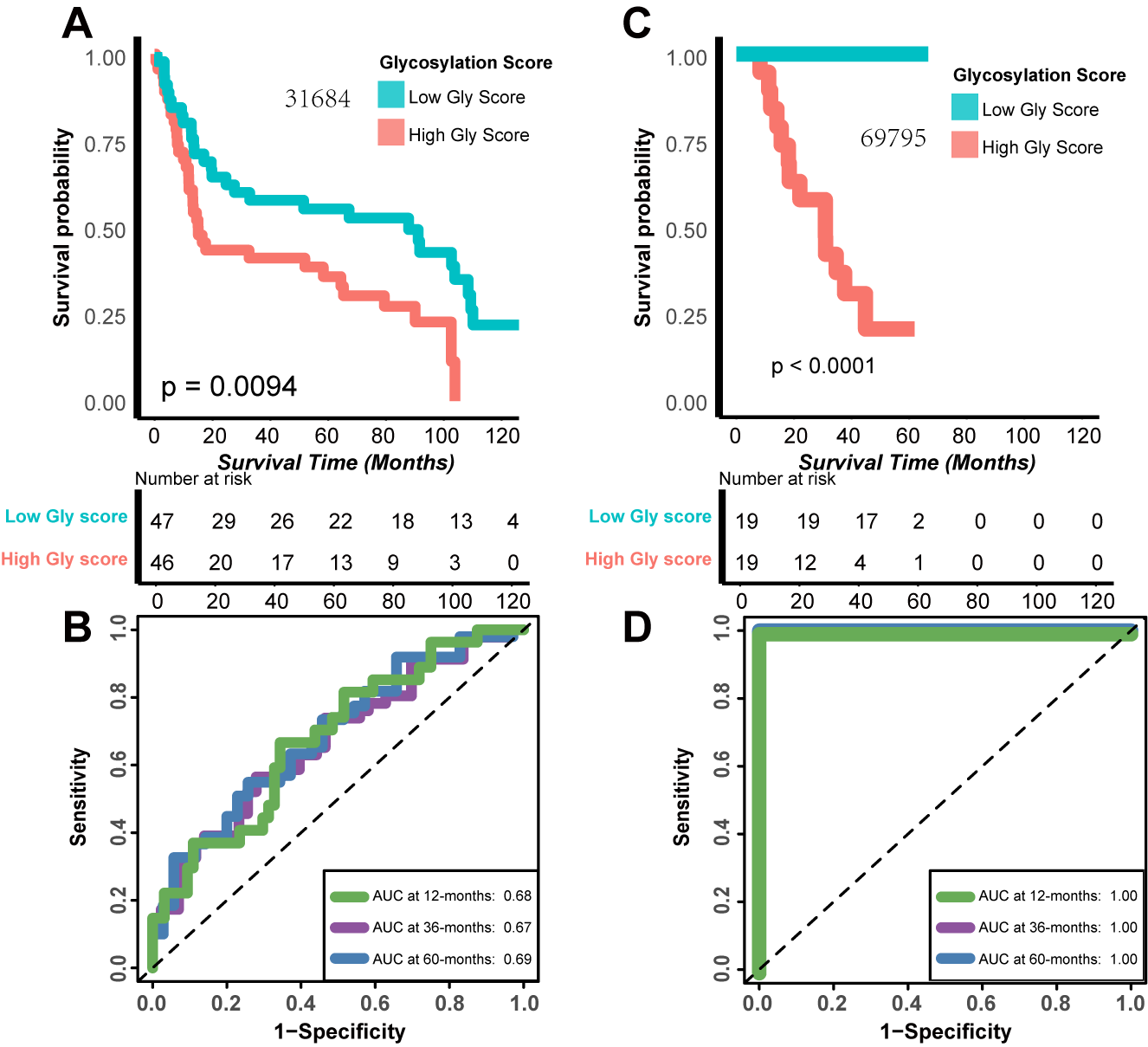

Supplement: Supplementary file 6 [file Image1.TIF]
